# A network analysis of dietary patterns, social participation patterns, and cognitive function in Chinese older adults

**DOI:** 10.3389/fpsyt.2026.1830990

**Published:** 2026-07-08

**Authors:** Yudie Zhang, Fang Yang, Jun Wu, Fei Shen, Yan Shen, Jianfen Ma, Xiaodong Cao

**Affiliations:** Affiliated Wuxi People’s Hospital of Nanjing Medical University, Wuxi People’s Hospital, Wuxi Medical Center, Nanjing Medical University, Wuxi, China

**Keywords:** cognitive function, dietary patterns, network analysis, older adults, social participation patterns

## Abstract

**Background:**

Social participation and dietary patterns have been associated with cognitive function in older adults, and potential interactions may exist between social and behavioral factors. Understanding these associations may help identify potential targets for prevention and intervention efforts. Therefore, this study aimed to examine the network relationships among dietary patterns, social participation patterns, and cognitive function domains, and to visualize these complex associations using network analysis.

**Methods:**

A total of 9,539 adults aged ≥60 years were included from the Chinese Longitudinal Healthy Longevity Survey (CLHLS, 2017–2018). Cognitive function was assessed using the Chinese version of the Mini-Mental State Examination (MMSE). Dietary patterns and social participation patterns were derived based on commonly consumed foods and social activities. Restricted cubic spline (RCS) analysis was conducted to analyze the dose-response relationships. Network analysis was conducted to identify central and bridging nodes, and network stability was evaluated using case-dropping bootstrap procedures.

**Results:**

Network analysis revealed that M3 (Attention and Calculation), M5 (Language), and M1 (Orientation) showed the highest expected influence, while S3 (Leisure and Entertainment-based Social Participation), M3 (Attention and Calculation), and S1 (Housework-related Social Participation) showed the highest bridge strength. The correlation stability coefficient of the centrality, expected influence, and bridge strength is 0.75, indicating that the network exhibits good stability. RCS analyses revealed both linear and nonlinear dose–response relationships between lifestyle factors and cognitive function domains, with stronger effects observed at lower exposure levels for certain variables.

**Conclusions:**

This exploratory study identified complex associations among dietary patterns, social participation patterns, and cognitive function domains in Chinese older adults. These findings provide a basis for future longitudinal and interventional research.

## 1 Introduction

Cognitive function encompasses multiple domains, including orientation, attention, memory, language, and executive function, and plays a critical role in maintaining independence, quality of life, and healthy aging among older adults (1). Age-related declines in cognitive function are common and may adversely affect daily functioning and well-being. Cognitive impairment and dementia represent more severe manifestations of cognitive decline and constitute major public health challenges worldwide (2). According to the Global Dementia Observatory (GDO), approximately 55.2 million people worldwide are currently living with dementia, and this number is projected to increase to 78 million by 2030 and 139 million by 2050 (3). Given the importance of cognitive function for healthy aging and independent living, identifying potentially modifiable factors associated with cognitive health has become an important public health priority.

Social participation has been widely recognized as an important factor associated with cognitive function in later life. A longitudinal cohort study conducted in Japan reported that sustained social participation was associated with a reduced risk of dementia among older adults (4). Similarly, a systematic review and meta-analysis of longitudinal studies showed that formal social participation was associated with reduced cognitive decline, although with low certainty of evidence (5). More recently, Abaei et al. reported dynamic longitudinal associations between loneliness and cognitive function in older adults, suggesting that social experiences and cognitive health may influence one another over time (6). Furthermore, evidence from a six-year longitudinal study demonstrated that maintaining a high level of social participation or increasing participation over time was associated with a significantly lower risk of cognitive impairment compared with persistently low participation levels (7). These findings further support the potential role of social participation in promoting cognitive health among older adults.

Dietary patterns have also been associated with cognitive functioning in older adults. Evidence from prospective cohort studies indicates that adherence to a healthy plant-based dietary index (hPDI) is associated with a lower risk of cognitive impairment and dementia (8). A narrative review of longitudinal studies conducted in Japan reported that a nutritionally balanced diet, integrating a variety of foods and nutrients, can be associated with the maintenance of brain function (9). More recently, Dempsey et al. found that higher adherence to the Mediterranean-DASH Intervention for Neurodegenerative Delay (MIND) diet was associated with better memory performance and appeared to attenuate the negative impact of cerebrovascular pathology on cognitive functioning in older adults at elevated dementia risk (10). Collectively, these findings suggest that dietary patterns may play an important role in maintaining cognitive health among older adults. These associations may operate through multiple biological and behavioral pathways, including inflammation, vascular health, oxidative stress, and metabolic regulation (11).

Although previous studies have examined associations between dietary patterns and cognitive function or between social participation and cognitive outcomes, most have focused on pairwise relationships using traditional variable-centered approaches. Consequently, little is known about how dietary patterns, social participation patterns, and specific cognitive function domains are interconnected within an integrated system. Furthermore, emerging evidence suggests that dietary behaviors and social participation may themselves be related, indicating that social and behavioral factors may not operate independently (12). More recently, evidence from a 10-year longitudinal study demonstrated that unhealthy plant-based dietary patterns and social isolation frequently co-occurred and were jointly associated with adverse aging outcomes (13). In addition, social support has been reported to be positively associated with dietary quality among community-dwelling older adults (14). Therefore, exploring the network relationships among social participation patterns, dietary patterns, and cognitive function domains is essential, as it may help identify central and bridge components within the system and inform targeted intervention strategies for cognitive health.

Network analysis has been increasingly applied in recent years to investigate complex relationships among psychological, behavioral, and health-related variables (15–17). It conceptualizes observable variables—such as attitudes, behaviors, and functional domains—as nodes, and uses regularization techniques to estimate partial correlation networks among these variables (18). This approach enables the examination of the structural organization of interrelated variables and the identification of clusters of closely connected nodes (19, 20).

Within such networks, central nodes are those that exhibit stronger connections with other nodes, indicating their prominent positions within the overall network structure (21, 22). However, it is important to note that centrality reflects statistical interconnectedness rather than causal importance. Additionally, network analysis can identify bridge nodes that connect different communities or domains (23). These bridge nodes may help to characterize how different domains are statistically linked within the network, providing insights into the overall structure of associations (24).

Unlike traditional approaches that primarily focus on isolated associations between lifestyle factors and cognitive outcomes, network analysis enables the simultaneous examination of complex interrelationships among multiple domains and the identification of central and bridge nodes within the system. Therefore, the present study applied a network analytic framework to examine the relationships among dietary patterns, social participation patterns, and cognitive function domains in Chinese older adults. Specifically, we aimed to identify central and bridge nodes within the network and to characterize the structural relationships among dietary patterns, social participation patterns, and cognitive function domains.

## 2 Method

### 2.1 Source of data and participants

The cross-sectional data were derived from the 2017–2018 wave of the Chinese Longitudinal Healthy Longevity Survey (CLHLS). The CLHLS was organized by the Center for Healthy Aging and Development Studies (CHADS) at Peking University and represents one of the first nationwide longitudinal studies of older adults in developing countries. The CLHLS employed a multistage, stratified cluster sampling design and covered approximately 85% of the Chinese population across 23 provinces, autonomous regions, and municipalities in mainland China. Between 1998 and 2018, the CLHLS was conducted in seven waves with a cumulative sample size of 15,874 participants (25).

All CLHLS data are publicly available through the Peking University Open Research Data Platform (https://opendata.pku.edu.cn/dataverse/CHADS). The survey collected detailed information on participants’ demographic characteristics, family background, socioeconomic status, self-rated health and quality of life, psychological characteristics, activities of daily living, lifestyle behaviors, and dietary habits.

**Figure 1:**
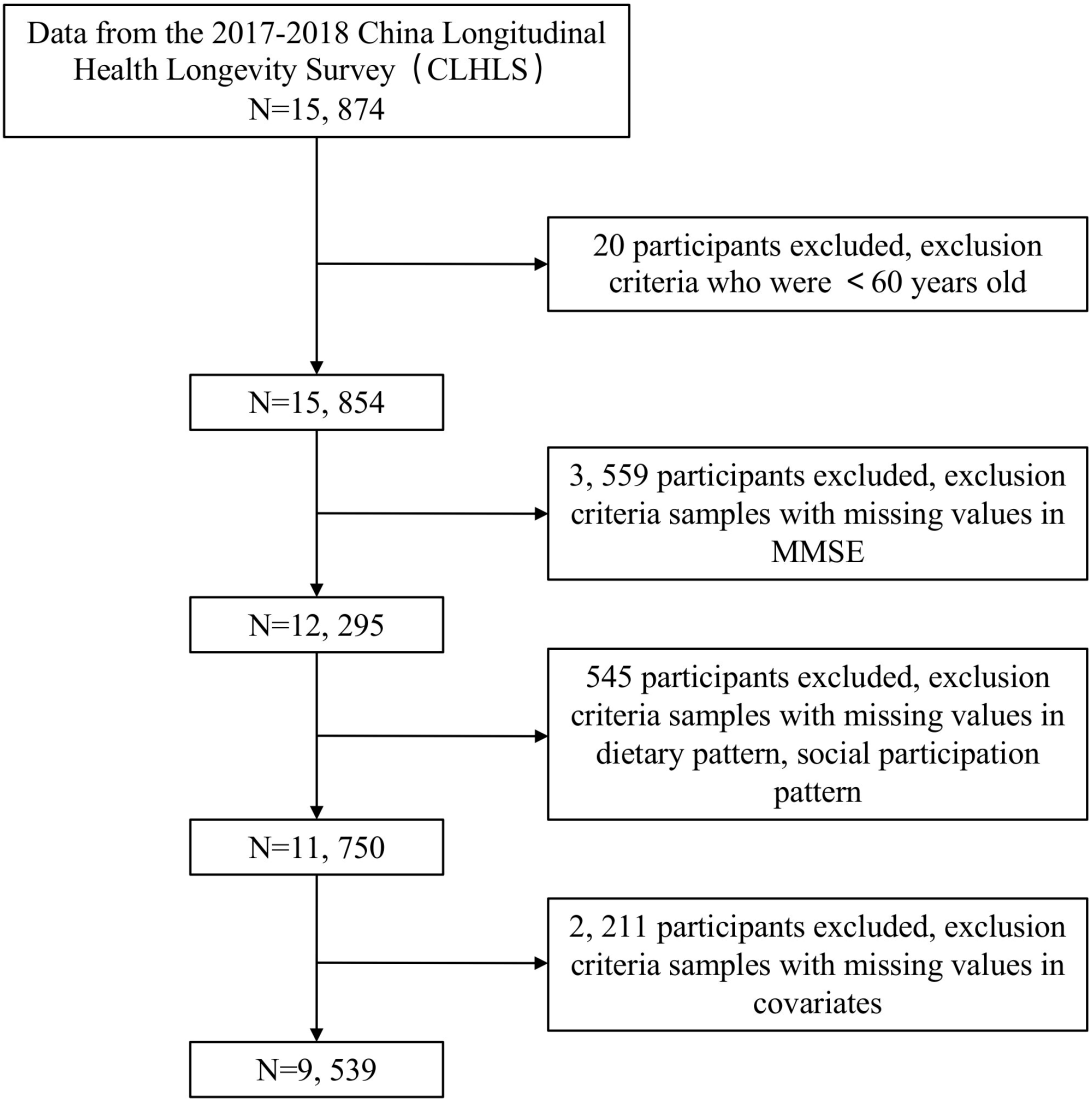
Flowchart of the participants.

For the present study, participants were included if they: (1) were aged 60 years or older; (2) completed the Chinese version of the MMSE; and (3) provided complete information on dietary patterns, social participation, and covariates. Participants with missing data on cognitive function, dietary patterns, social participation patterns, or covariates were excluded from the analysis. The participant selection flow is presented in **Figure 1**. A total of 9,539 participants were included in the final analysis. The final sample included older adults from both urban and rural areas and represented diverse socioeconomic and geographic backgrounds across China. Detailed demographic and socioeconomic characteristics of the study participants are presented in **Table 1**.

**Table 1:** Basic characteristics of participants.

Variables		Number (%)
Age	60-79	3982 (41.74%)
	≥80	5557 (58.26%)
Sex	Male	4297 (45.05%)
	Female	5242 (54.95%)
Marital status	Having spouse	4502 (47.20%)
	No spouse	5037 (52.80%)
Place of residence	Urban	5561 (58.30%)
	Rural	3978 (41.70%)
Education	Illiterate	4281 (44.88%)
	Primary	3226 (33.82%)
	Junior high school or higher	2032 (21.30%)
Living standard	Poor	926 (9.70%)
	Average	6717 (70.42%)
	Rich	1896 (19.88%)
Life satisfaction	Satisfied	6713 (70.38%)
	Neutral	2551 (26.74%)
	Dissatisfied	275 (2.88%)
Health satisfaction	Satisfied	4604 (48.26%)
	Neutral	3718 (38.98%)
	Dissatisfied	1217 (12.76%)

The Human Subjects Protection Plan for the CLHLS was approved by the Duke University Research Ethics Committee (Pro00062871) and Peking University (IRB00001052-13074). Prior to the survey, written informed consent was obtained from all CLHLS participants.

### 2.2 Measurement

#### 2.2.1 Cognitive function

Cognitive function was assessed using the Chinese version of the Mini-Mental State Examination (MMSE). The Chinese version of the MMSE has demonstrated good validity and reliability in previous studies (24). The MMSE includes 24 items covering five cognitive domains: orientation (six items), responsiveness (three items), attention and calculation (six items), recall (three items), and language (six items).

In addition, item 6 was adapted into a one-minute food-naming task, in which one point was awarded for each correctly named food item, with a maximum score of seven points. The total MMSE score ranged from 0 to 30, with one point assigned for each correct response and zero points for incorrect answers. Cognitive impairment was defined as a score of or below 24 (26). The MMSE domains used in this study represent relatively broad indicators of cognitive function and may not capture subtle impairments across specific neuropsychological domains.

#### 2.2.2 Assessment of dietary patterns

To assess dietary patterns more accurately, food categories were selected based on evidence from previous studies. Nine food categories—fresh fruits, vegetables, meat, aquatic products (e.g., fish), eggs, soy products, dairy products, nuts, and tea—were selected (27). Based on previous research (28), responses regarding the frequency of intake for each food item were coded as 1 if participants reported consuming the food “almost daily” or “at least once per week”, and 0 otherwise. The total dietary pattern score ranged from 0 to 9, with higher scores indicating healthier dietary behaviors. Additionally, dietary patterns were categorized into animal-based and plant-based dietary patterns. The animal-based dietary pattern included meat, fish, eggs, and dairy products (score range: 0–4), whereas the plant-based dietary pattern included fruits, vegetables, soy products, nuts, and tea (score range: 0–5).

#### 2.2.3 Assessment of social participation patterns

The CLHLS database includes 11 items assessing social participation: engaging in housework, practicing Tai Chi, participating in square dancing, engaging in outdoor activities, visiting and interacting with friends, gardening (planting flowers and growing plants), reading books/newspapers, raising poultry/livestock, playing cards or mahjong, watching TV/listening to the radio, and participating in organized social activities. Each item was rated on a five-point scale (1–5), with higher scores indicating more frequent participation. In this study, social participation activities were categorized into three patterns (29): housework-related social participation (two items), social interaction-based social participation (three items), and leisure and entertainment-based social participation (six items). To minimize cumulative scoring effects, the highest score among the items within each pattern was used as the final score representing that type of social participation (30). However, this approach may oversimplify participation patterns.

#### 2.2.4 Assessment of covariates

Demographic variables collected included age (65–79 or ≥80 years), sex (male or female), marital status (with spouse or without spouse), education (illiterate, primary, or junior high school or higher), place of residence (rural or urban), living standard (poor, average, or rich), life satisfaction (satisfied, neutral, or dissatisfied), and health satisfaction (satisfied, neutral, or dissatisfied). These covariates were selected based on prior literature and their potential associations with cognitive function (24).

### 2.3 Statistical analysis

First, descriptive statistics for categorical variables were calculated using SPSS version 26.0, reporting frequencies and percentages. Second, Restricted cubic spline (RCS) analyses were performed using logistic regression models with the “lrm()” function in the “rms” package to evaluate potential dose–response relationships between dietary pattern scores, social participation pattern scores, and cognitive impairment. Models with 3, 4, and 5 knots were compared using the Akaike information criterion (AIC), and the model with the lowest AIC was selected. Odds ratios (ORs) and 95% confidence intervals (CIs) were estimated after adjustment for age, sex, marital status, education, place of residence, living standard, life satisfaction, and health satisfaction.

Third, network analysis was conducted using R (version 4.5.2). The network model linking dietary patterns, social participation patterns, and cognitive function domains was estimated using the EBICglasso method implemented in the R package “bootnet”. The network was visualized using the “qgraph” package, with nodes representing variables and edges representing regularized partial correlations between nodes. Edges were colored to indicate the direction of associations (blue for positive and red for negative), and edge thickness reflected the strength of the associations (23).

A Gaussian Graphical Model (GGM) was applied, and the graphical least absolute shrinkage and selection operator (glasso) was used to estimate a sparse and regularized network. The Extended Bayesian Information Criterion (EBIC) was used to select the optimal tuning parameter for regularization (20).

Node centrality was primarily assessed using expected influence (EI), which is considered more appropriate than traditional strength centrality in networks containing both positive and negative edges (31). Higher EI values indicate greater overall connectivity of a node within the network.

Bridge centrality was estimated using the “networktools” package. Bridge strength was calculated to identify nodes that connect different predefined communities. Communities were defined based on conceptual domains, including cognitive function, dietary patterns, and social participation patterns.

Additionally, the “bootnet” package was used to assess the accuracy and stability of the network. A nonparametric bootstrap procedure was performed to estimate the 95% confidence intervals (CIs) of edge weights, with narrower CIs indicating greater accuracy. The stability of centrality indices was evaluated using the correlation stability coefficient (CS-C), where values above 0.25 indicate acceptable stability, values above 0.50 indicate good stability, and values above 0.70 indicate high stability (32).

RCS analysis was conducted to examine dose–response relationships with cognitive impairment, while network analysis was used to explore the structural associations among cognitive function domains and related factors.

## 3 Results

### 3.1 Descriptive results

**Table 1** presents the detailed demographic characteristics of the participants. The participants included 9,539 older adults, with a mean age of 82.99 (SD = 11.33) years: 4,297 males and 5,242 females.

4,502 (47.20%) of the older adults had a spouse, 4,281 (44.88%) were illiterate, and 3,978 (41.70%) lived in rural areas. 926 (9.70%) of the older adults had a poor living standard, 6,713 (70.38%) reported being satisfied with their life, and 4,604 (48.26%) reported being satisfied with their health. .

### 3.2 Correlation analysis

**Figure 2:**
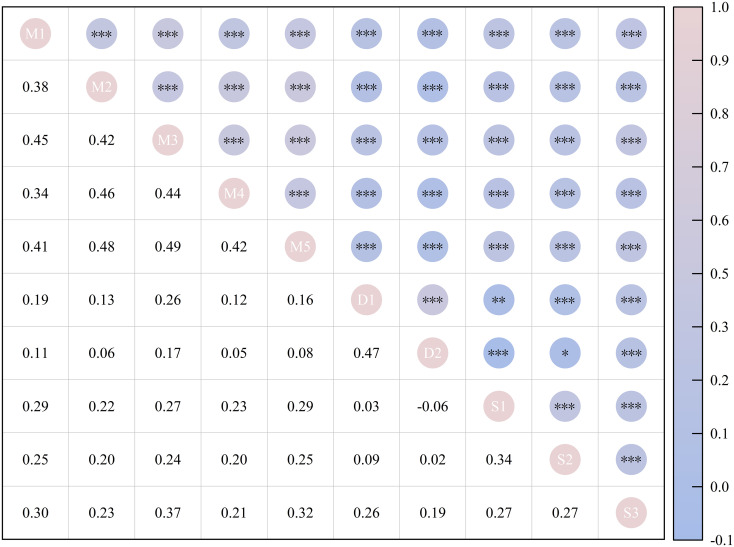
Correlation analysis of cognitive function, dietary patterns, and social participation patterns. * P < 0.05, ** P <0 .01, *** P < 0.001

**Figure 2** illustrates the correlations between cognitive function, dietary patterns, and social participation patterns of the participants. Significant associations were found between dietary patterns, social participation patterns, and all domains of cognitive function: Orientation, Responsiveness, Attention and Calculation, Recall, and Language, with varying magnitudes.

### 3.3 Dose-response analysis

**Figure 3:**
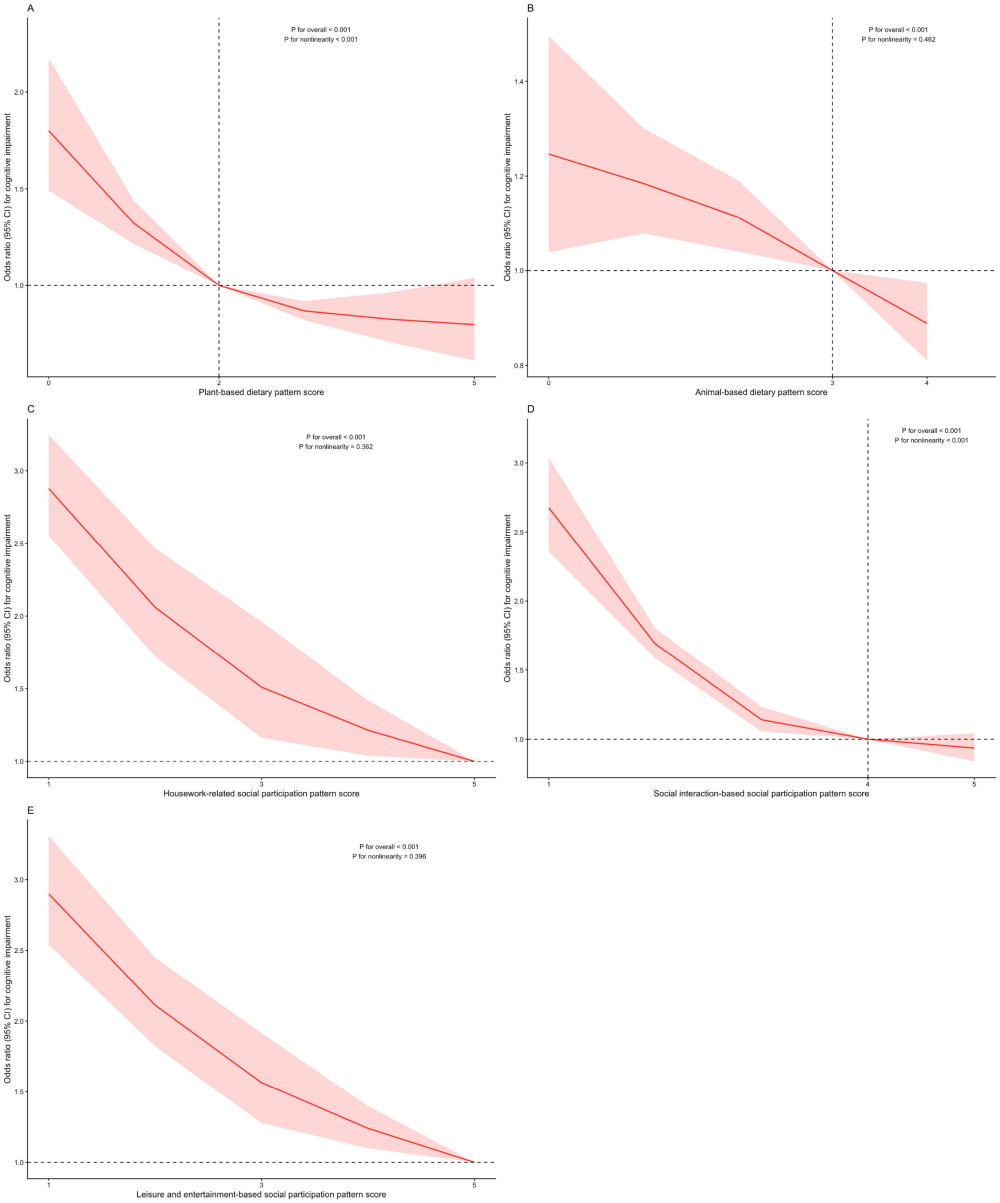
Dose–response relationships between dietary patterns, social participation patterns, and cognitive impairment based on restricted cubic spline models. Panel **(A)** shows the association between plant-based dietary pattern score and cognitive impairment; Panel **(B)** shows the association for animal-based dietary pattern score; Panel **(C)** for housework-related social participation pattern score; Panel **(D)** for social interaction-based social participation pattern score; and Panel **(E)** for leisure and entertainment-based social participation pattern score. The red solid line represents the estimated odds ratios, and the shaded area indicates the 95% confidence intervals. The horizontal dashed line represents an odds ratio of 1.0, and the vertical dashed line indicates the reference value. All models were adjusted for age, sex, marital status, education, place of residence, living standard, life satisfaction, and health satisfaction.

Restricted cubic spline analyses revealed significant dose–response relationships between dietary patterns, social participation patterns, and cognitive impairment. The results of the dose–response analysis are presented in **Figure 3**. Plant-based dietary pattern score showed a significant nonlinear association with cognitive impairment (P for overall < 0.001; P for nonlinearity < 0.001). The risk of cognitive impairment decreased sharply at lower levels of plant-based dietary scores and then gradually plateaued.

Animal-based dietary pattern score was linearly associated with cognitive impairment (P for overall < 0.001; P for nonlinearity = 0.462), suggesting that higher scores were associated with lower odds of cognitive impairment.

Similarly, housework-related social participation pattern score demonstrated a linear inverse association with cognitive impairment (P for overall < 0.001; P for nonlinearity = 0.362). Leisure and entertainment-based social participation pattern score also showed a linear inverse relationship (P for overall < 0.001; P for nonlinearity = 0.396).

Social interaction-based social participation pattern score exhibited a significant nonlinear association (P for overall < 0.001; P for nonlinearity < 0.001), with a steep reduction in risk at lower levels followed by a plateau at higher levels.

### 3.4 Analysis of network structure and centrality measures

**Figure 4:**
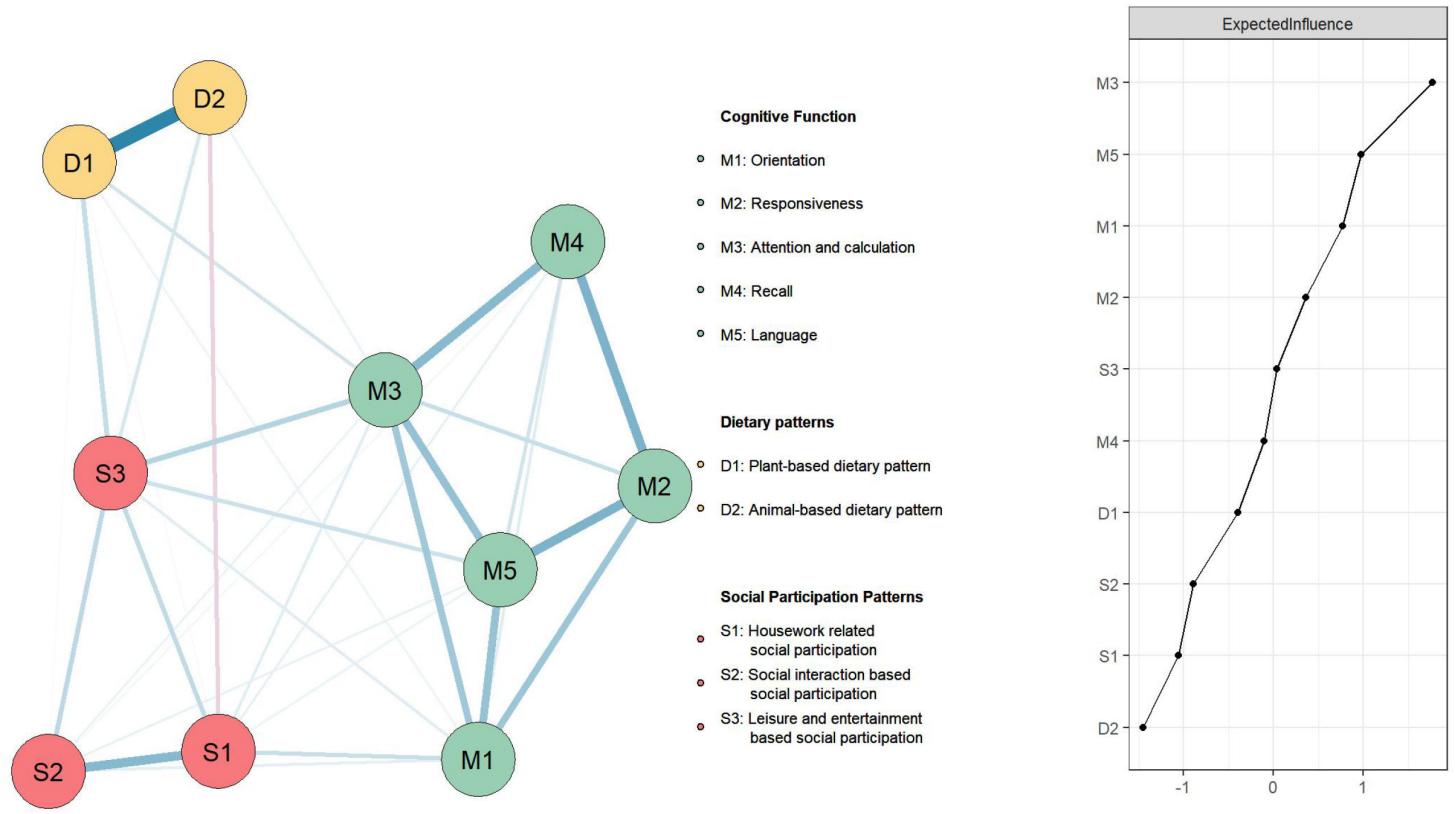
Network structure of dietary patterns, social participation patterns, and cognitive function domains in older adults. Nodes represent variables, and edges represent regularized partial correlations estimated using EBICglasso. Blue edges indicate positive associations, while red edges indicate negative associations. Edge thickness reflects the strength of the associations.

The left subgraph in **Figure 4** illustrates the network structure of dietary patterns, social participation patterns, and cognitive function, with a network density of 0.82 (37/45) and an average edge weight of 0.08. 10 nodes generated 45 potential edges, of which 37 edges had a non-zero weight (82.22%). The correlation matrix is shown in **Supplementary Table 1**. The strength of the connections is represented by edge thickness and color intensity. Within the cognitive subnetwork, the strongest association was between M2 (Responsiveness) and M4 (Recall), followed by M2 (Responsiveness) and M5 (Language). Within the social participation subnetwork, the strongest association was between S1 (Housework-related social participation) and S2 (Social interaction-based social participation). Between the dietary, social participation, and cognitive function networks, M3 (Attention and Calculation) and S3 (Leisure and entertainment-based social participation) were strongly associated, followed by D1 (Plant-based dietary pattern) and S3 (Leisure and entertainment-based social participation), and M1 (Orientation) and S1 (Housework-related social participation).

The right subgraph of **Figure 4** shows that, within the cognitive subnetwork, M3 (Attention and Calculation) had the highest expected influence (EI), whereas M4 (Recall) had the lowest EI. In the dietary subnetwork, D1 (Plant-based dietary pattern) had the highest EI, while D2 (Animal-based dietary pattern) had the lowest EI. In the social participation subnetwork, S3 (Leisure and entertainment-based social participation) had the highest EI, while S1 (Housework-related social participation) had the lowest EI.

In the full network model, M3 (Attention and Calculation) showed the highest expected influence, followed by M5 (Language), M1 (Orientation), and M2 (Responsiveness). In contrast, D2 (Animal-based dietary pattern) showed the lowest expected influence. The centrality difference results (**Supplementary Figure 1**) showed that these nodes showed differences compared with other nodes.

### 3.5 Bridge network structure and bridge strength analysis

**Figure 5:**
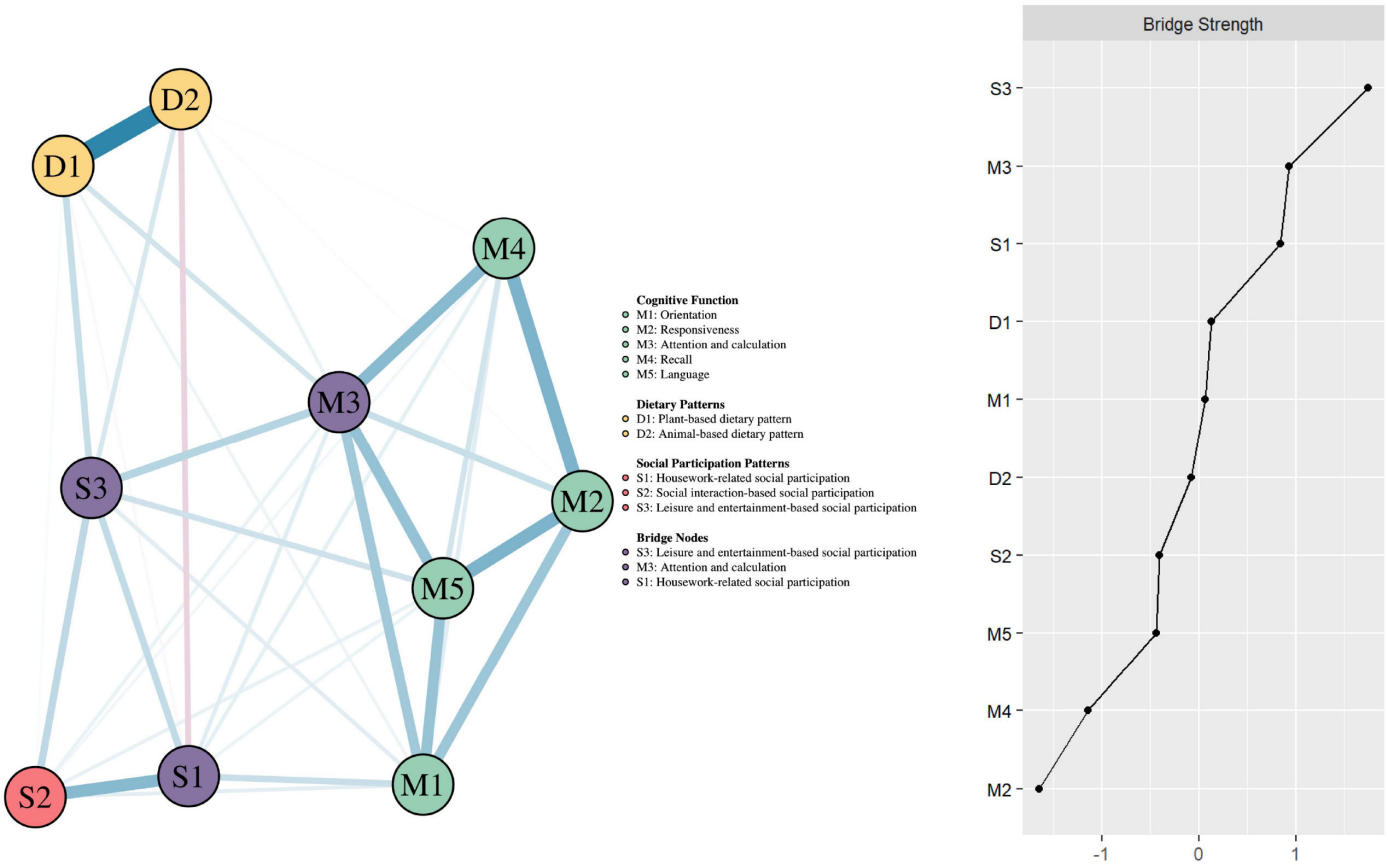
Network with highlighted bridge nodes and bridge strength centrality. Nodes with higher bridge strength are highlighted, indicating their roles in connecting different domains within the network. The panel on the right displays the standardized bridge strength values of each node. Blue edges indicate positive associations, while red edges indicate negative associations.

**Figure 5** shows the bridge strength of the network. S3 (Leisure and entertainment-based social participation) had the highest bridge strength, followed by M3 (Attention and Calculation) and S1 (Housework-related social participation), suggesting that these nodes may play important roles in linking different domains within the network. The bridge centrality difference test (**Supplementary Figure 2**) showed differences in bridge strength among nodes, providing additional support for the robustness of the identified bridge nodes.

### 3.6 Network stability

**Figure 6:**
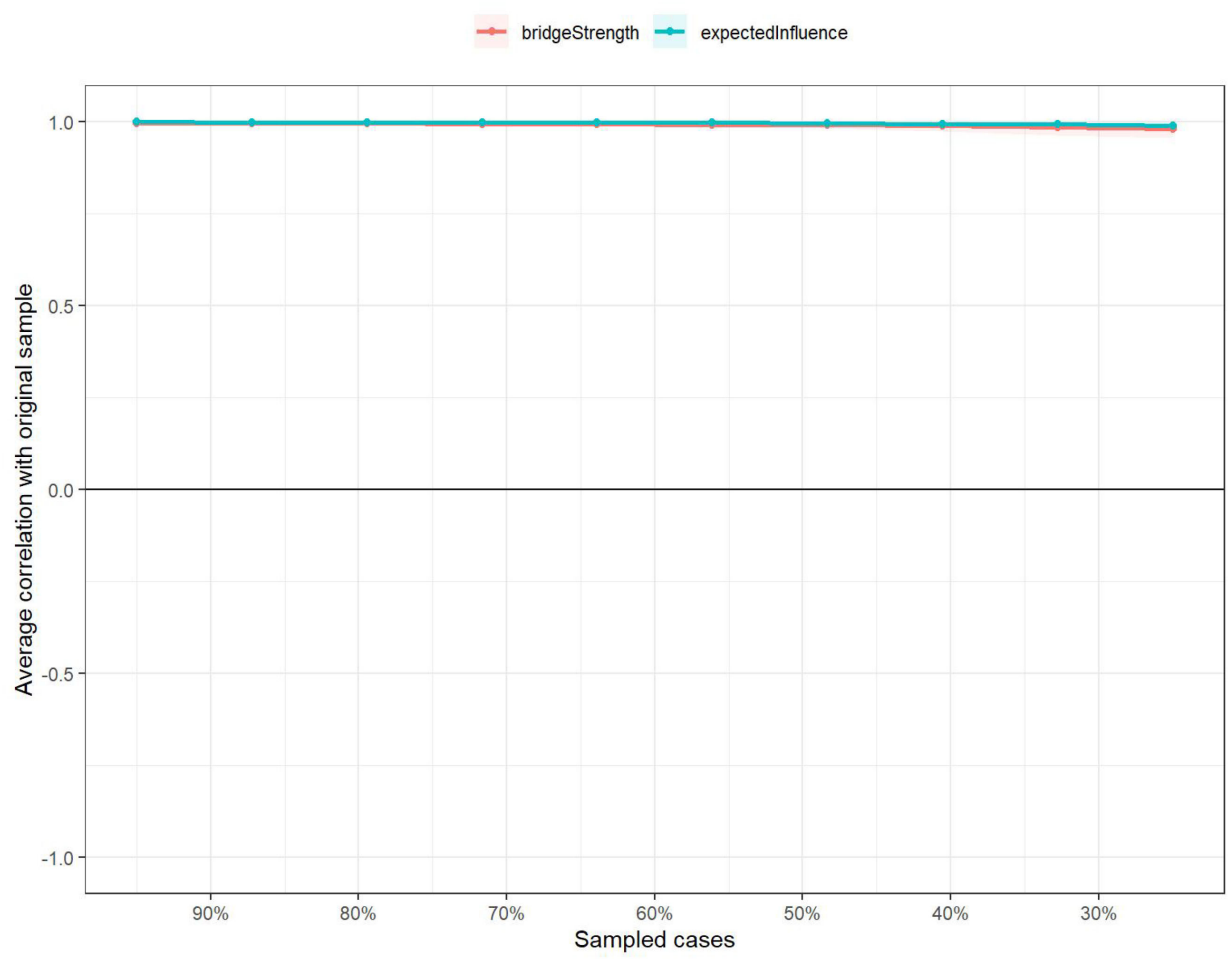
Stability of expected influence and bridge strength estimated using case-dropping bootstrap. The x-axis represents the proportion of the sample retained, and the y-axis represents the correlation between centrality indices from the original network and those from re-estimated networks based on subsamples. The lines indicate the stability of expected influence and bridge strength across different sampling levels.

**Supplementary Figure 3** examines the accuracy of the marginals using bootstrap 95% confidence intervals, indicating acceptable accuracy of edge-weight estimates. **Figure 6** presents the results of the case-dropping bootstrap test. The CS coefficient of centrality (EI) was 0.75, indicating that the original network and the re-estimated order of centrality (EI) remained consistent at 0.75 when 75% or fewer of the sample size were excluded. The CS coefficient for bridge strength was 0.75, indicating that the bridge strength was sufficiently stable.

## 4 Discussion

To our knowledge, this is among the first studies to simultaneously examine dietary patterns, social participation patterns, and cognitive function domains within an integrated network framework among Chinese older adults. A large sample of Chinese older adults was analyzed using network analysis to examine the interrelationships among dietary patterns, social participation patterns, and cognitive function domains. Within the network, M3 (Attention and Calculation), M5 (Language), and M1 (Orientation) demonstrated higher expected influence, indicating that these nodes were more strongly connected with other variables in the network. In addition, S3 (Leisure and entertainment-based social participation), M3 (Attention and Calculation), and S1 (Housework-related social participation) showed higher bridge strength, suggesting their potential roles as connectors across different domains. In this study, the term “bridge node” is used in a broader methodological sense to describe variables that connect different domains (i.e., dietary patterns, social participation, and cognitive function), rather than implying clinical symptom bridging as defined in psychiatric networks. Furthermore, D2 (Animal-based dietary pattern) was negatively associated with S1 (Housework-related social participation) among Chinese older adults.

M3 (Attention and Calculation) exhibited the highest expected influence within the network, indicating its central position in the overall network structure. Previous studies have highlighted the importance of attention-related functions in cognitive performance. For example, a study found that attention is one of the cognitive variables that best accounts for cognitive impairment, suggesting that attention tasks are effective tests for cognitive impairment screening purposes (33), findings from a study focusing on a 3-minute reaction time (RT) task suggest that reaction time deficits are a sensitive indicator of dementia (34). In addition, results from a randomized controlled trial (RCT) indicated that a 4-week simple arithmetic intervention had a positive effect on the cognitive function of older adults (35), another study found that learning tasks, such as arithmetic computation, contribute to cognitive rehabilitation in patients with dementia, potentially activating the bilateral dorsolateral prefrontal cortex (DLPFC) through arithmetic computation (36). These findings support the relevance of attention and calculation within broader cognitive functioning. However, it should be noted that centrality in network analysis reflects statistical interconnectedness rather than causal importance.

Bridge strength analysis identified S3 (Leisure and entertainment-based social participation) as a node with relatively strong connections across domains. In network analysis, bridge nodes may help to characterize how different domains are statistically linked, although they should not be interpreted as causal pathways (37). Previous studies have reported consistent associations between leisure activities and cognitive function (38, 39). For instance, a systematic review and meta-analysis indicated that engagement in cognitively stimulating leisure activities is associated with a lower risk of cognitive decline (40), and findings from a cross-sectional study conducted in China suggest that a higher frequency of participation in leisure activities is associated with better cognitive function. These findings further validate the credibility of our results (41). These findings are broadly consistent with our results.

Within the network, M3 (Attention and Calculation) and S3 (Leisure and entertainment-based social participation) showed relatively strong associations, suggesting a statistical association between cognitive function domains and leisure-related social participation. The Multi-level Leisure Mechanisms Framework identified over 600 types of leisure activities, and described the potential mechanisms underlying the effects of leisure activities on health and cognition (42). This framework suggests that leisure activities may be related to cognitive function through multiple pathways, including psychological, social, biological, and behavioral processes (42). These mechanisms not only exert direct effects on physical and mental health, but also interact across domains (e.g., bidirectional interactions between psychological and social mechanisms), providing a theoretical basis for understanding how leisure activities are associated with cognitive health (43). D1 (Plant-based dietary pattern) and S3 (Leisure and entertainment-based social participation) were also closely associated, consistent with the findings of previous studies. For example, a study conducted in the UK found that active participation in leisure activities was associated with higher diet quality (defined as frequent consumption of fruits, vegetables, grains, etc.) (12). Findings from a prospective cohort study conducted in Sweden indicated that an active lifestyle (including leisure-social activities) may be associated with more favorable cognitive outcomes in the context of healthy dietary patterns (44). However, these relationships should be interpreted as associative rather than causal.

D2 (Animal-based dietary pattern) was negatively associated with S1 (Housework-related social participation). Older adults who assume more household responsibilities are often family caregivers and may prioritize their family members’ dietary needs, preparing animal-based foods for their families first. Alternatively, those with insufficient family support tend to choose simple and affordable plant-based foods (45). These interpretations remain speculative and warrant further investigation.

This study has several strengths. First, it applies a network analysis framework to examine the complex interrelationships among dietary patterns, social participation patterns, and multiple domains of cognitive function, allowing for the identification of central and bridge nodes within the system. Second, the data sample derived from the CLHLS is large, representative, and stable. Third, this study integrates multiple lifestyle and cognitive dimensions within a unified analytical framework, providing a more comprehensive understanding of their interconnected structure. However, several limitations should be acknowledged. First, the cross-sectional study design precludes causal inference, and all findings should be interpreted as exploratory associations. Second, dietary patterns and social participation were measured using relatively simplified indicators, which may not fully capture the complexity of these constructs. Additionally, cognitive function was assessed using MMSE, a widely used screening instrument for global cognitive functioning. Although the MMSE allows assessment across several cognitive domains, including orientation, attention and calculation, recall, and language, it provides a relatively broad evaluation and may not capture subtle cognitive deficits in specific neuropsychological domains, such as executive functioning, verbal fluency, and delayed memory, with the same precision as comprehensive neuropsychological assessments. Third, potential residual confounding from unmeasured variables (e.g., depression, comorbidities, and functional status) cannot be excluded. Fourth, the findings of this study are based on a specific cultural and population context in China, and therefore should be interpreted within this setting. Differences in dietary habits, social structures, and lifestyle patterns across countries may limit the generalizability of these results to other populations. Future studies are needed to validate these findings in diverse cultural and geographic contexts.

In conclusion, this study revealed complex associations between dietary patterns, social participation patterns, and cognitive function domains among older adults in China using a network analysis approach. Nodes with higher expected influence and bridge strength may provide insights into the structural organization of these relationships. Future longitudinal and interventional studies are needed to further clarify the directionality and potential mechanisms underlying these associations.

## Data Availability

Publicly available datasets were analyzed in this study. This data can be found here: https://opendata.pku.edu.cn/dataset.xhtml?persistentId=doi:10.18170/DVN/WBO7LK&version=2.0.
