# Supplementary material for: A network analysis of dietary patterns, social participation patterns, and cognitive function in Chinese older adults
Source: Front Psychiatry. 2026 Jul 8;17:1830990. doi: 10.3389/fpsyt.2026.1830990 (PMC13389496; doi:10.3389/fpsyt.2026.1830990)
Supplement: Supplementary file 1 [file SupplementaryFile1.docx]

Supplementary Material

# Supplementary Figures and Tables.

## Supplementary Figures


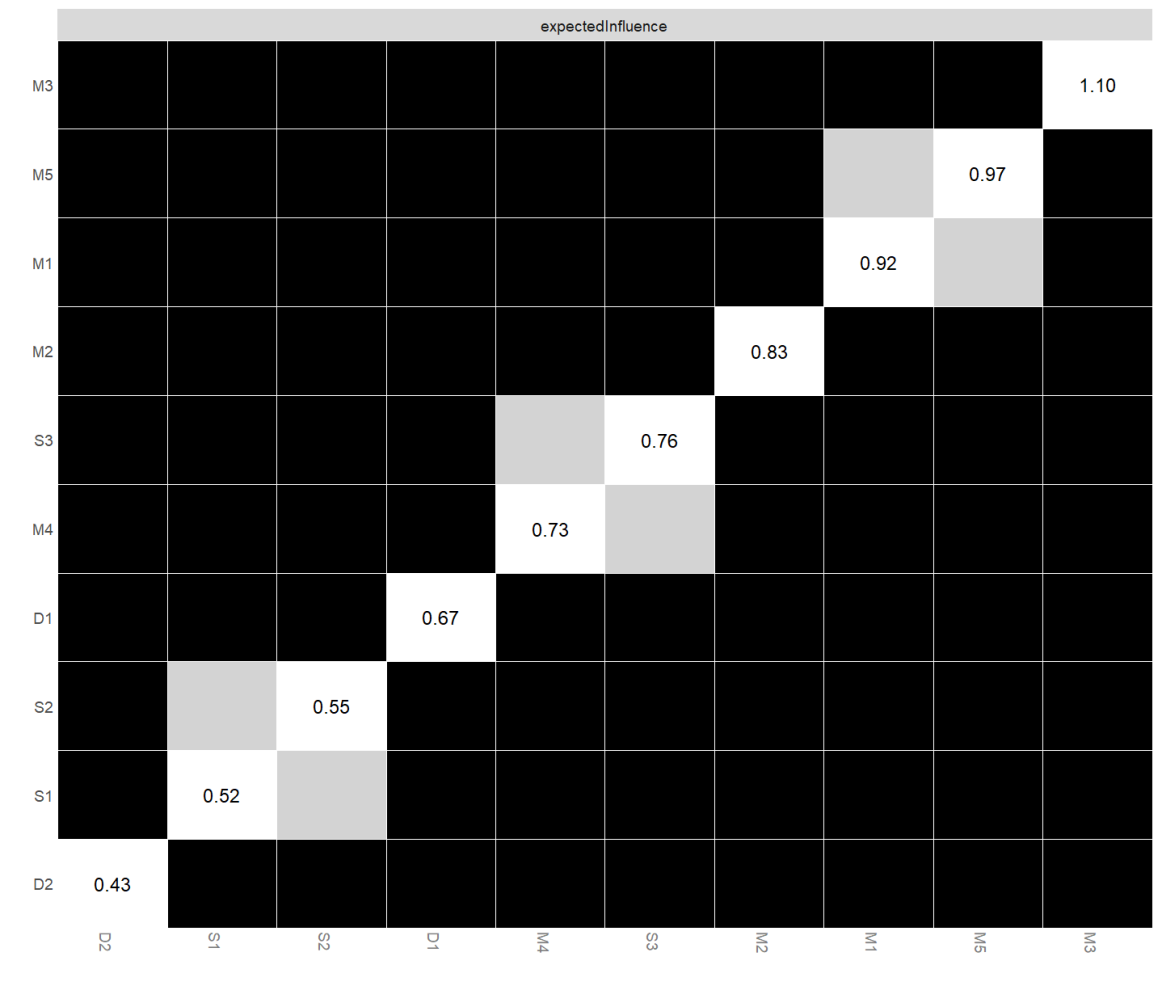


**Figure S1.** Bootstrapped difference test for EI of dietary patterns, social participation patterns and cognitive function. Note: Gray boxes indicate that there are no significant differences between nodesʹ expected influences, while black boxes indicate that there are significant differences between nodesʹ expected influences. The numbers in the white boxes (i.e., diagonal lines) indicate the numerical values of the nodesʹ expected influences.


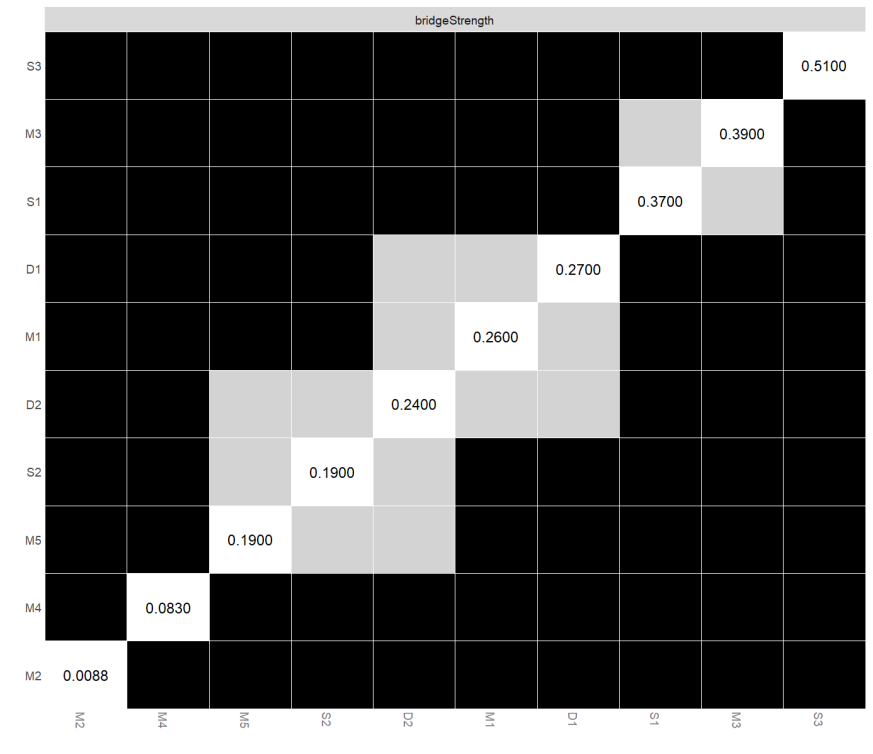


**Figure S2.** Bootstrapped difference test for bridge strength of dietary patterns, social participation patterns and cognitive function. Note: Gray boxes indicate that there are no significant differences between nodesʹ bridge strength, while black boxes indicate that there are significant differences between nodesʹ bridge strength. The numbers in the white boxes (i.e., diagonal lines) indicate the numerical values of the nodesʹbridge strength.


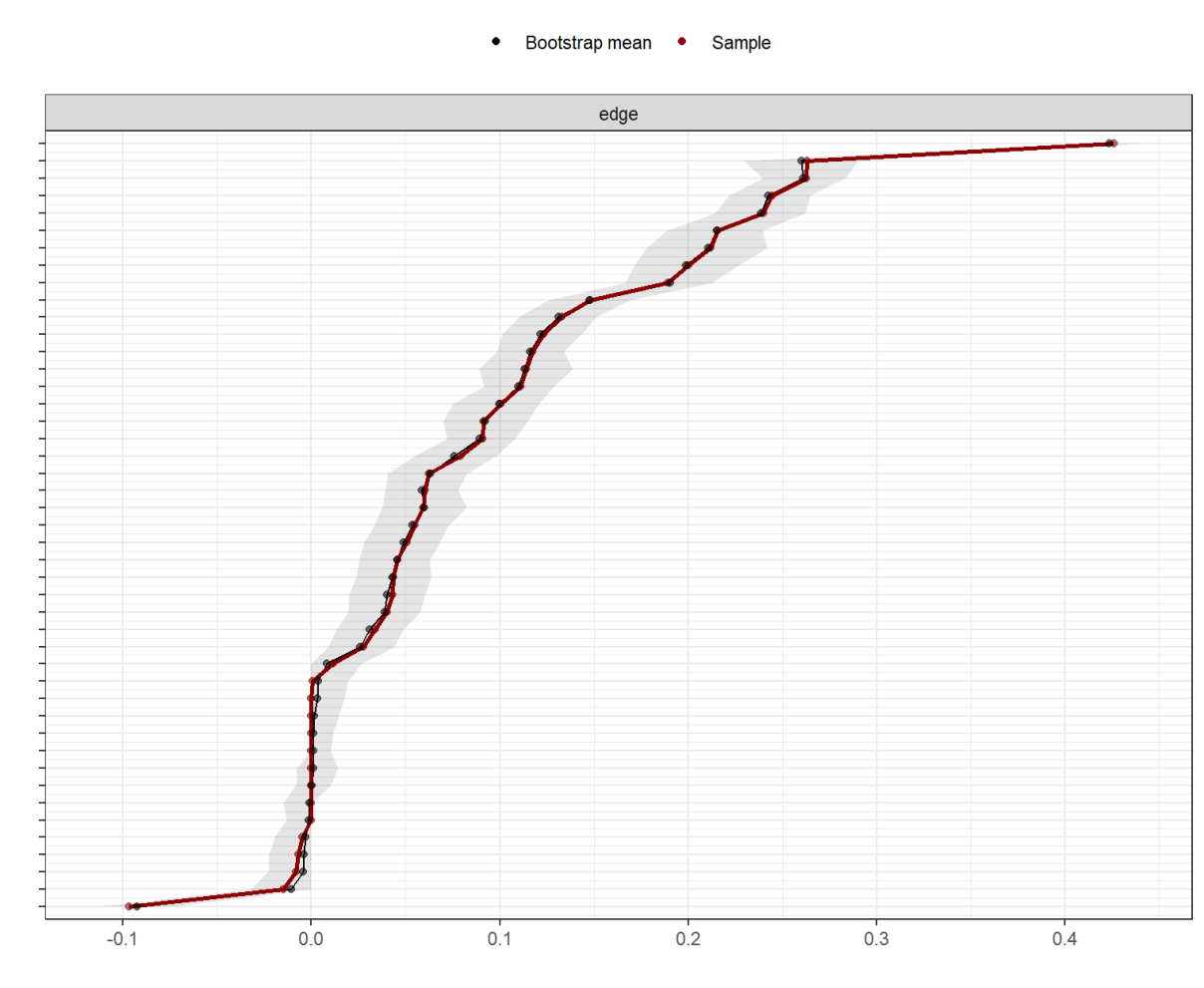


**Figure S3.** Bootstrapped confidence intervals of edge weights. Note: The red line indicates the edge estimated in the sample, and the black dot indicates the bootstrap mean for that edge. Gray areas represent 95% bootstrap confidence intervals for the edge, with narrower gray lines indicating more accurate estimates of the edge.

## Supplementary Tables

**Table S1.** Weighted adjacency matrix of dietary patterns, social participation patterns and cognitive function.

|  | M1 | M2 | M3 | M4 | M5 | D1 | D2 | S1 | S2 | S3 |
| --- | --- | --- | --- | --- | --- | --- | --- | --- | --- | --- |
| M1 | 1.000 |  |  |  |  |  |  |  |  |  |
| M2 | 0.500 | 1.000 |  |  |  |  |  |  |  |  |
| M3 | 0.517 | 0.671 | 1.000 |  |  |  |  |  |  |  |
| M4 | 0.435 | 0.731 | 0.590 | 1.000 |  |  |  |  |  |  |
| M5 | 0.490 | 0.728 | 0.720 | 0.640 | 1.000 |  |  |  |  |  |
| D1 | 0.182 | 0.209 | 0.295 | 0.162 | 0.240 | 1.000 |  |  |  |  |
| D2 | 0.084 | 0.101 | 0.198 | 0.076 | 0.125 | 0.505 | 1.000 |  |  |  |
| S1 | 0.382 | 0.402 | 0.359 | 0.338 | 0.471 | 0.042 | -0.067 | 1.000 |  |  |
| S2 | 0.309 | 0.343 | 0.299 | 0.281 | 0.400 | 0.113 | 0.029 | 0.451 | 1.000 |  |
| S3 | 0.344 | 0.408 | 0.498 | 0.328 | 0.507 | 0.338 | 0.246 | 0.397 | 0.391 | 1.000 |
